# Large Volume Direct Injection Ultra-High Performance Liquid Chromatography–Tandem Mass Spectrometry-Based Comparative Pharmacokinetic Study between Single and Combinatory Uses of *Carthamus tinctorius* Extract and Notoginseng Total Saponins

**DOI:** 10.3390/pharmaceutics12020180

**Published:** 2020-02-20

**Authors:** Jinfeng Chen, Xiaoyu Guo, Yingyuan Lu, Mengling Shi, Haidong Mu, Yi Qian, Jinlong Wang, Mengqiu Lu, Mingbo Zhao, Pengfei Tu, Yuelin Song, Yong Jiang

**Affiliations:** 1 State Key Laboratory of Natural and Biomimetic Drugs, School of Pharmaceutical Sciences, Peking University, Beijing 100191, China; chenjinfeng0513@163.com (J.C.); guoxiaoyu@bjmu.edu.cn (X.G.); luyingyuan2005@126.com (Y.L.); shimenglingbj@126.com (M.S.); a1538933944@163.com (H.M.); yiqian@163.com (Y.Q.); wang-jinlong@pku.edu.cn (J.W.); mengqiulu@163.com (M.L.); zmb_77@163.com (M.Z.); pengfeitu@bjmu.edu.cn (P.T.); 2 Modern Research Center for Traditional Chinese Medicine, Beijing University of Chinese Medicine, Beijing 100029, China

**Keywords:** *Carthamus tinctorius* extract, notoginseng total saponins, comparative pharmacokinetic study, large volume direct injection, compatibility mechanism

## Abstract

The combination of *Carthamus tinctorius* extract (CTE) and notoginseng total saponins (NGTS), namely, CNP, presents a synergistic effect on myocardial ischemia protection. Herein, comparative pharmacokinetic studies between CNP and CTE/NGTS were conducted to clarify their synergistic mechanisms. A large volume direct injection ultra-high performance liquid chromatography–tandem mass spectrometry (LVDI-UHPLC-MS/MS) platform was developed for sensitively assaying the multi-component pharmacokinetic and in vitro cocktail assay of cytochrome p450 (CYP450) before and after compatibility of CTE and NGTS. The pharmacokinetic profiles of six predominantly efficacious components of CNP, including hydroxysafflor yellow A (HSYA); ginsenosides Rg_1_ (GRg_1_), Re (GRe), Rb_1_ (GRb_1_), and Rd (GRd); and notoginsenoside R_1_ (NGR_1_), were obtained, and the results disclosed that CNP could increase the exposure levels of HSYA, GRg_1_, GRe, GRb_1_, and NGR_1_ at varying degrees. The in vitro cocktail assay demonstrated that CNP exhibited more potent inhibition on CYP1A2 than CTE and NGTS, and GRg_1_, GRb_1_, GRd, quercetin, kaempferol, and 6-hydroxykaempferol were found to be the major inhibitory compounds. The developed pharmacokinetic interaction-based strategy provides a viable orientation for the compatibility investigation of herb medicines.

## 1. Introduction

Myocardial ischemia-induced infarction is one of the leading causes of human death worldwide. The benefits of either *Carthamus tinctorius* extract (CTE) or notoginseng total saponins (NGTS) towards myocardial ischemia injury on rats have been well defined, and more interestingly, previous studies have demonstrated that better cardio-protective effects were observed when using their combination preparation (CNP) [1,2,3]. However, the underlying synergetic mechanisms of CTE and NGTS combination, their pharmacokinetic (PK) interactions in particular, still remain unclear.

It is widely accepted that the drug–drug interactions (DDIs) and herb–herb interactions (HHIs) can cause changes of pharmacokinetic profiles, which result in the possible improvement of drug efficacy and in the decrease of side effects, or vice versa [4,5]. However, most of the literature has merely focused on the pharmacokinetic profile variations of these primary components between individual dosing and combined use, but has overlooked the reasons responsible for the changed pharmacokinetic behaviors, which may be caused, at least in part, by cytochrome p450 (CYP450)- and/or transporter-mediated HHIs [6]. Therefore, the objective of this study was to gain insight into the synergistic actions between CTE and NGTS by determination of the pharmacokinetic profiles of six major active components from CTE and NGTS, as well as their CYP450-based synergetic mechanisms. An in vitro cocktail assay, which is an efficient and widely favored approach for CYP450-mediated HHIs, was employed to pursue the factors accounting for the different pharmacokinetic patterns before and after compatibility.

Our previous pharmacological evaluations optimized a relatively low dosage CNP for the anti-myocardial ischemia effect [2]. Furthermore, the cocktail method usually suffers from extensive CYP450 crossover within the probe substrates [7]. Therefore, the emerging demand is to develop a sensitive and efficient method for reliable detection and determination of the trace ingredients for the PK and cocktail studies. Attempts were made herein to propose and apply a large volume direct injection ultra-high performance liquid chromatography–tandem mass spectrometry (LVDI-UHPLC-MS/MS) method for direct and sensitive multiple-component PK and cocktail studies.

## 2. Materials and Methods

### 2.1. Plant Materials

**Figure 1:**
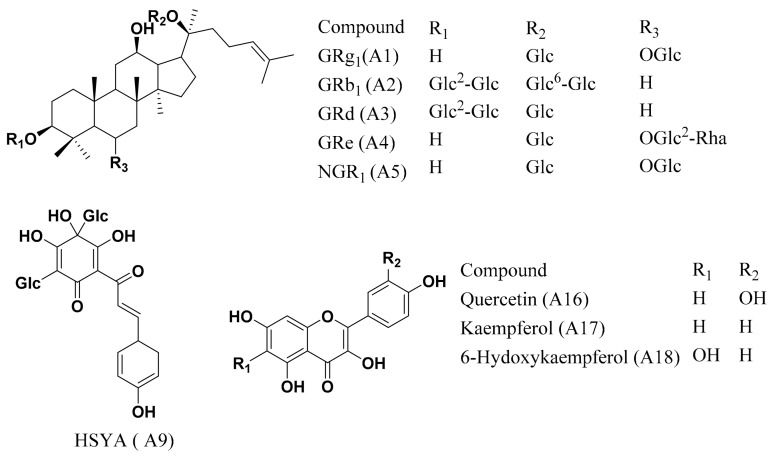
The chemical structures of the main components contained in the combination of *Carthamus tinctorius* extract and notoginseng total saponins (CNP).

Notoginseng total saponins (NGTS), containing ginsenoside Rg_1_ (GRg_1_, A_1_, 26.6%), ginsenoside Rb_1_ (GRb_1_, A2, 32.5%), ginsenoside Rd (GRd, A3, 6.6%), ginsenoside Re (GRe, A4, 4.1%), and notoginsenoside R_1_ (NGR_1_, A5, 6.2%), prepared according to the Monograph of NGTS recorded in Chinese Pharmacopoeia [8], was purchased from Yunnan Plant Pharm. Co., Ltd. (Yunnan, China). Besides NGR_1_, GRg_1_, GRe, GRb_1_, and GRd (Han et al., 2010), the remaining amount of around 25% ginsenosides in NGTS were further clarified as previously described [9]. *Carthamus tinctorius* extract, containing hydroxysafflor yellow A (HSYA; A9, 8.0%) and kaempferol-3-*O*-rutinoside (A11, 0.2%) was prepared following the protocol described in a previous report [1]. The chemical structures of the main components contained in CNP are shown in Figure 1. The chemical profiling based on LC–MS (Figure S1) and the detail chemical composition information were reported in our previous research papers [10]. The detailed information of other chemicals and reagents is shown in the Supplementary Materials section.

### 2.2. Animals and Rat Liver Microsomes

Male Sprague-Dawley (SD) rats (12–14 weeks, 200–240 g) were provided by the Experimental Animal Center, Peking University Health Science Center. All animal experimental protocols were approved by the Biomedical Ethical Committee of Peking University Health Science Center (SYXK (Jing) 2016-0041, 23 December 2016). The animal experiments were carried out in accordance with the National Institutes of Health guide for the care and use of laboratory animals (NIH Publications no. 8023, revised 1978).

Pooled SD rat liver microsome (RLM, 20 mg/mL, LM-DS-02M, BDVH) were purchased from the Research Institute for Liver Diseases (Shanghai, China) Co. Ltd.

### 2.3. Plasma Pharmacokinetic Studies

CTE, NGTS, and CNP powders were dissolved using saline, and were orally dosed at 50 mg·kg^−1^, 60 mg·kg^−1^, and 50 mg·kg^−1^ CTE + 60 mg·kg^−1^ NGTS, respectively, whereas saline was administered to the vehicle group. Six rats were used in each group, and all rats fasted overnight but had free access to water prior to treatment. A terminal sampling design was used to collect blood samples at 0 (pre-dose), 0.08, 0.16, 0.25, 0.5, 1, 2, 3, 4, 6, 8, 12, 24, 48, 72, and 96 h. At each time, 0.5 mL of blood was collected in the heparin sodium tubes. Plasma was separated by centrifugation at 4000 rpm for 10 min and stored below −80 °C until bioanalysis. Oasis PRiME HLB SPE cartridges (1 cc/30 mg, Waters, Milford, MA, USA) were used to process the plasma samples (Supplementary Materials).

### 2.4. Incubation Procedure and CYP450 Activity Assay

The effects of CTE, NGTS, CNP, and 18 representative compounds (A1–A18, Supplementary Materials) on CYP450 activities were investigated using a pool of SD RLM following the procedure in [11]. Incubations were conducted at 37 ± 1 °C in 200 μL of incubation mixtures containing RLM (0.2 mg/mL), phosphate buffer saline (PBS) (pH 7.4, 0.1 mM), MgCl_2_ (5 mM), nicotinamide adenine dinucleotide phosphate (NADPH) (1 mM), and CYP450 probe substrates (90/1.07/18/0.13/0.02/3.6/90 μM of phenacetin/omeprazole/ tolbutamide/dextromethorphan/midazolam/chlorzoxazone/ bupropion, respectively). CYP450 inhibitors (triethylenethiophosphoramide/sulfaphenazole/ticlopidine/furafylline/ ketoconazole/quinidine/4-methylpyrazole for CYP2B6/2C9/2C19/1A2/3A4/2D6/2E1, respectively) were added as the positive control, and blank solvents (PBS containing methanol and/or dimethyl sulfoxide (DMSO)) were used as the negative control. The incubation mixtures also contained eight concentrations for CTE (2.5–200 µg/mL), NGTS (2.5–200 µg/mL), CNP (2.5–200 µg/mL), A1–A17 (5–200 μM), and A18 (0.25–10 µM). Reactions were initiated by adding the NADPH-generating system and terminated after 15 min by 200 μL of cold methanol containing 105 nM hydroxybupropion-[D_6_] (OHBUP-[D_6_]) and 5 nM 4′-hydroxydiclofenac-[^13^C_6_] (OHDIC-[^13^C_6_]) as internal standards (ISs). The mixture was placed in an ice bath for 30 min, and the precipitated protein was removed by centrifugation (12,000 rpm for 10 min at 4 °C) three times. Then an aliquot of 100 μL supernatant was diluted with 100 µL ultrapure water, and centrifuged at 12,000 rpm for 10 min, before being subjected to LVDI-UHPLC-MS/MS analysis.

### 2.5. LVDI-UHPLC-MS/MS Analysis

**Figure 2:**
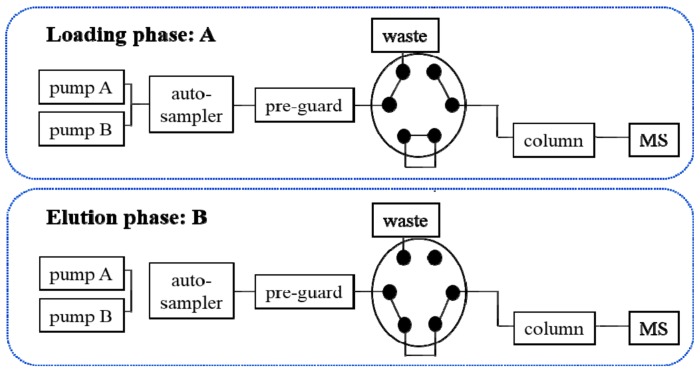
Connectivity sketch of the six-port/two-channel switching valve controlling the large volume direct injection ultra-high performance liquid chromatography–tandem mass spectrometry (LVDI-UHPLC-MS/MS) system. At the loading phase, the valve was maintained at A-channel. The sample delivered from the auto-sampler was captured onto a pre-guard column. Meanwhile, the pumps delivered the mobile phase at a high flow rate. Then, the valve was automatically switched to the B-channel to trigger the elution phase. The trapped components were flushed from the pre-guard column into an analytical column.

The generic layout of instrumentation setup of the LVDI-UHPLC-MS/MS was conducted on a Shimadzu UHPLC system consisting of two LC-20ADXR pumps, a DGU-20A3R degasser, and a CBM-20A controller (Kyoto, Japan) with a SCIEX 4500 QTRAP mass spectrometer mounted with an electron spray ionization (ESI) interface as well as an electronic 6-port/2-channel valve (Foster City, CA, USA). Analyst software package (version 1.6.2, SCIEX) was implemented to control and synchronize the entire system, and also for data acquisition and processing. A single analytical run was fragmented into two phases, namely, loading and elution phases, by switching the electronic valve (Figure 2). At the loading phase, the valve was maintained at A-channel. The specimen aliquot of large volume delivered from the auto-sampler was captured onto a guard column. Meanwhile, the pumps were responsible for delivering the mobile phase at a high flow rate, aiming to dilute the sample solvent, thus facilitating the candidate constituents to concentrate on the pre-guard column. Then, the valve was automatically switched to the B-channel to trigger the elution phase. The trapped components were flushed from the pre-guard column into an analytical column, and underwent multiple reaction monitoring (MRM) analysis on the optimized LC gradients. The detailed information of LVDI-UHPLC-MS/MS analysis for pharmacokinetic and cocktail studies is shown in the Supplementary Materials section.

### 2.6. Method Validations

Method validations for the pharmacokinetic and cocktail assays, in terms of specificity, linearity, and sensitivity; precision and accuracy; recovery and matrix effect; and stability, were individually carried out by following the U.S. Food and Drug Administration (FDA) Guidance on Bioanalytical Method Validation and Drug Interaction Studies [12].

### 2.7. Data Processing

Calibration data were fitted to linear calibration curves using 1/x^2^ weighting. For the PK study, the half-time (*T*_1/2_), maximum plasma concentration (*C*_max_), time to reach the maximum concentration (*T*_max_), and area under concentration-time curve (AUC) were determined by non-compartmental method using Drug and Statistics 3.0 (DAS 3.0, Mathematical Pharmacology Professional Committee of China, Shanghai, China). For the cocktail assay, the activity is expressed as the percentage of activity remaining comparing with that of a control sample containing no inhibitor. Substrate inhibition data were analyzed using GraphPad Prism 6 (version 6.01; San Diego, CA, USA) in logistic regression. All the data were described as mean ± standard deviation (SD). Normality assumptions were tested by the Kolmogorov–Smirnov statistic setting *p* = 0.10 as the limit for rejection of the null hypothesis of normality. If the distribution of the data was normal with equal variances, a two-sided test was performed at the 5% level of significance. The Welch’s correction was then applied when the underlying variances were not equal. When the assumption of normality must be rejected, the Mann-Whitney test, a non-parametric equivalent of the independent-measures *t*-test, was used.

## 3. Results

### 3.1. Injection Solvent Optimization

It is well known that the sample solvent and volume have large effects on the peak asymmetry and column efficiency [13]. Herein, we established a LVDI-UHPLC-MS/MS method, assisted by injection solvent optimization, for sensitive bioassays of the pharmacokinetic interactions and cocktail analysis of CTE and NGTS. Considering the solubility and the sample pretreatment methods (Supplementary Materials) of the target analytes, the mixed standard solution of the six reference compounds was diluted with methanol (MeOH)/water (H_2_O) in a ratio of 20% increment ranging from 100% H_2_O to 100% MeOH for the PK study, and all the six standards of the PK study showed the highest responses when using 40% or 60% aqueous MeOH as the injection solvent. The CYP450 probe substrates and their corresponding metabolites for the in vitro cocktail assay were chosen according to the FDA guide [14]. Likewise, the six metabolites for the cocktail assay showed the best chromatograms when the 25% aqueous MeOH was served as the sample solvent, with exception of 1′-hydroxymidazolam, for which 50% aqueous MeOH was selected as the injection solvent (Figure S2).

### 3.2. Optimization of the Loading Phase for the LVDI-UHPLC-MS/MS-Based Method

The instrument stability of the LVDI-UHPLC-MS/MS setup was first investigated, and the results indicated the LVDI-UHPLC-MS/MS could meet the demands of quantitation (Table S3). An analytical run of the LVDI-UHPLC-MS/MS-based method was fragmented into a loading phase and an elution phase. The gradient condition for the elution phase turned out to be the same as that of the regular UHPLC-MS/MS analysis. Thus, the optimization works were concentrated on the loading phase, including the flow rate, mobile phase, and dilution time. According to the optimized gradient programs of the elution phases, both PK and cocktail studies employed water as the mobile phase for their loading phase after evaluating the solvents ramped from 0% to 20% aqueous acetonitrile. The flow rate of the loading phase for the PK study was finally optimized as 3.0 mL·min^−^^1^ after assays of 0.4, 1.0, 2.0, and 3.0 mL·min^−^^1^ flow rates. Because the higher the flow rate, the lower the signal responses of paracetamol and 6-hydroxychlorzoxazone, the cocktail assay finally chose 0.4 mL·min^−1^ as the flow rate of the loading phase. It turned out that the dilution time did not have a profound effect on the total chromatogram by comparing 0.5, 1, and 2 min. Thus, the shortest time, 0.5 min, was chosen to promote the analytical efficiency. The corresponding bioanalytical method validations were carried out by following the FDA guidance [12], and the results demonstrated that the newly developed LVDI-UHPLC-MS/MS-based methods enabled reliable detection and precise determination of the multiple-component in PK and cocktail studies. The lower limits of quantitation (LLOQ) of most analytes (except Rb_1_ and Rd) were lower than 60 pg/mL.

### 3.3. Comparative Multiple-Component PK Studies

**Figure 3:**
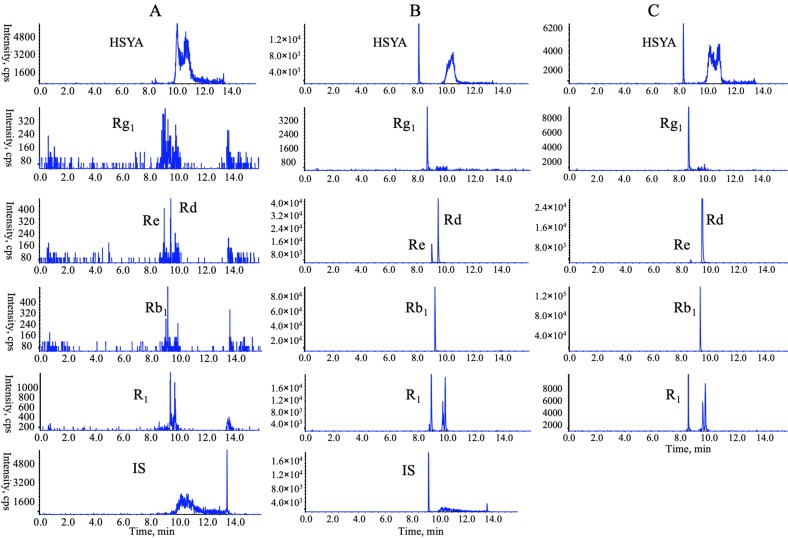
Representative multiple reaction monitoring (MRM) chromatograms of target analytes in rat plasma in a positive mode: (**A**) blank plasma; (**B**) blank plasma spiked with six chemical standards and internal standards (IS); (**C**) plasma sample collected at 2 h following oral administration of extract CNP (*Carthamus tinctorius* extract (CTE) 50 mg/kg + notoginseng total saponins (NGTS) 60 mg/kg) to rats.

**Figure 4:**
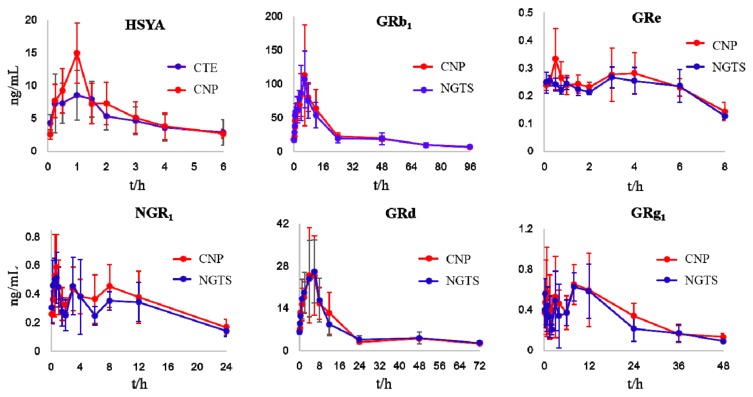
Mean plasma concentration-time profiles of the six analytes in rats after oral administration of CTE, NGTS, and CNP. Each point represents the mean ± SD (*n* = 6).

Considering the bioavailability improvement of active ingredients is a key point of traditional Chinese medicine compatibility, we executed a multiple-component PK study. HSYA, GRb_1_, GRd, GRe, GRg_1_, and NGR_1_ were the primary circulating compounds and the main cardio-protective components in CNP [15,16], and thus were chosen as the PK markers. Meanwhile, the optimized injection solvent and LVDI-UHPLC-MS/MS method were integrated to achieve a much more sensitive method for reliable quantification of these components in vivo. After validation (Figure 3, Tables S4–S7), the developed method was first applied to characterize the pharmacokinetic characters of HSYA, GRb_1_, GRd, GRe, GRg_1_, and NGR_1_ in rat plasma. Their plasma concentrations versus time profiles are displayed in Figure 4 and Table S8. The combination group showed greater *C*_max_ and AUC_0-t_ values (Table 1) of HSYA, GRg_1_, GRb_1_, NGR_1_, and GRe over the individual extract groups. Exceptionally, GRd exhibited considerable AUC_0-t_ and *C*_max_ between NGTS and CNP dosing groups, whereas significantly different *T*_1/2_ values, which indicated the combination use of CTE and NGTS, may have accelerated the elimination processes of GRd. The reason may have been due to the hydrolysis of GRb_1_ to GRd in vivo [17], resulting in a more complicated PK behavior of GRd than other compounds in CNP.

### 3.4. CYP450-Mediated Herb–Herb Interactions

**Figure 5:**
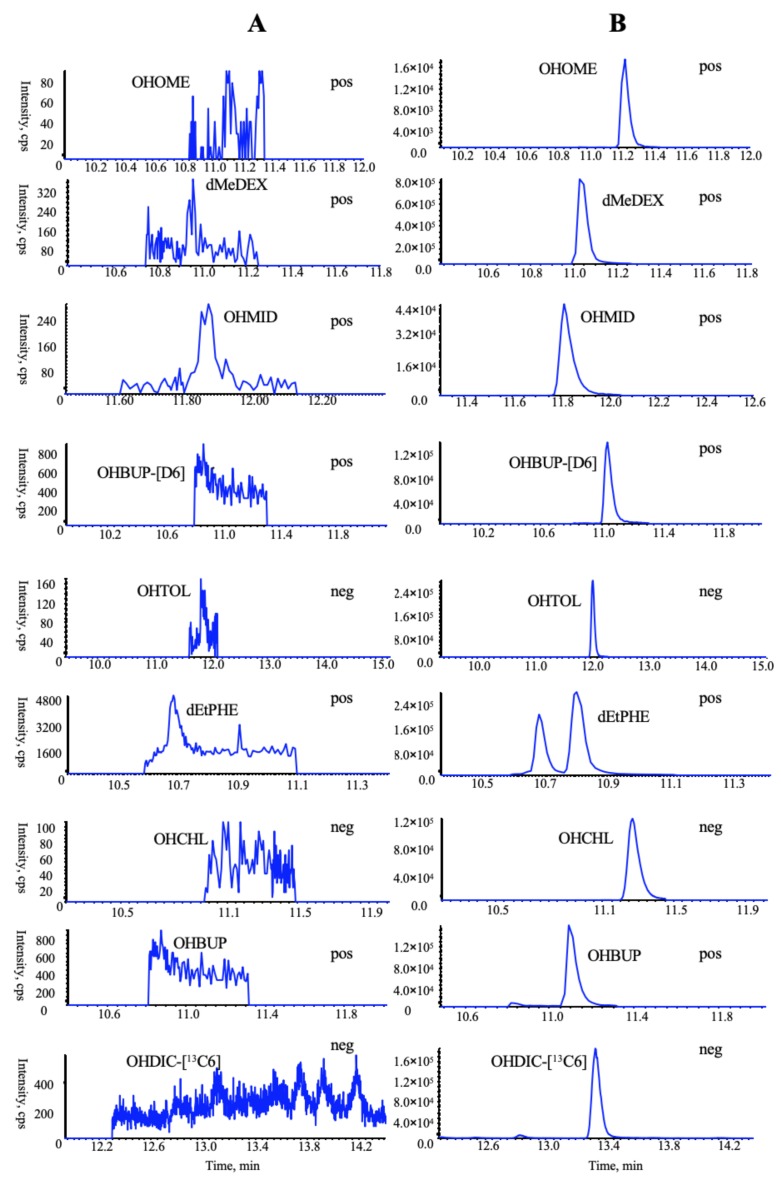
Representative MRM chromatograms of all probe metabolites and two ISs in the incubated rat microsomal sample with no substrate cocktails: (**A**) blank microsomal sample, (**B**) blank microsomal sample spiked with seven chemical standards and two ISs monitored in a polarity switching mode.

To investigate the possible HHIs between NGTS and CTE, an in vitro cocktail assay involving seven probe substrates was conducted, with the assistance of LVDI-UHPLC-MS/MS method to avoid CYP450 crossovers (Figure 5, Tables S9–S11). The incubation system was optimized in the aspects of substrate choice, enzyme concentration, incubation time, and substrate concentration (Figures S3–S4, Table S12), following the guidance of the FDA [14]. According to the half inhibiting concentration (IC_50_) values of CTE, NGTS, and CNP (Table 2), CTE showed weak inhibition on CYP1A2, CYP2D6, and CYP2C9 and moderate inhibition on CYP2B6 and CYP2E1, whereas NGTS presented much more potent inhibitions on all these detected CYP450s than CTE (Table 2). After combination, CNP showed more potent inhibition on CYP1A2 than CTE and NGTS, and more potent inhibition on CYP2C9, CYP2C19, CYP3A4, and CYP2D6 than CTE (Table 2).

To identify the major CNP components responsible for the inhibition, 18 representative components from CNP were evaluated by the cocktail assays. The results (Table 2) showed that HSYA and ginsenosides showed high or moderate inhibition activities on the seven CYP450 isozymes. Although flavonoid glycosides showed moderate or weak inhibition activities, or even no inhibition activities (IC_50_ values of >200 μM, Table 2), the flavonoid aglycones quercetin (A16), kaempferol (A17), and 6-hydoxykaempferol (A18), in contrast, exhibited potent inhibitory activities against the seven CYP450 isozymes. In particular, 6-hydroxykaempferol (A18) showed remarkably stronger inhibitory activities on the seven CYP450 isozymes (IC_50_ < 5 μM, Table 2). In conclusion, the ginsenosides GRg_1_ (A1), GRb_1_ (A2), and GRd (A3), and the flavonoids 6-hydoxykaempferol-3-*O*-glucoside (A6), kaempferol-3-*O*-glucoside (A7), anhydroxysafflor yellow B (AHSYB, A8), hydroxysafflor yellow A (HSYA, A9), 6-hydroxykaempferol-3,6-di-*O*-glucoside (A13), quercetin (A16), kaempferol (A17), and 6-hydroxykaempferol (A18) were presented as being the intensive inhibitors to different CYP450s (IC_50_ ≤ 15 μM). Among them, GRg_1_ (A1), GRb_1_ (A2), GRd (A3), quercetin (A16), kaempferol (A17), and 6-hydroxykaempferol (A18) were the main active components of CNP for the inhibition of CYP1A2.

### 3.5. Discussion

Herbal pair, the most fundamental and simplest form of Chinese herbal medicine formula, has been favored for centuries because of its better therapeutic outcomes and fewer side effects [18]. Herein, we primarily aimed to clarify the compatibility mechanisms between NGTS and CTE from PK interactions. Given the low dosage, more efforts were paid onto the detection and quantification of trace CNP-derived components in vivo. We found the injection solvent extensively affected the chromatographic performances. Increasing the injection volume could advance the sensitivity owing to the subjection of larger amounts of analytes [19]. However, the solvent effect might be initiated by directly injecting large volume of solution onto the chromatographic column without any additional treatment. Increasing the flow rate of the mobile phase could guarantee the dilution of the injection solvent and retention of the target analytes, which should be a practical choice to minimize, or even avoid the solvent effect. However, a rapid flow rate gave rise to a higher back pressure. Therefore, the evaporation–reconstitution step often involved loading the sufficient quantity of sample onto the column for LC–MS analysis. Fortunately, the electronic six-port/two-channel valve mounted on the QTRAP system can be applied as a viable solution to split the back pressure. Therefore, LVDI-UHPLC-MS/MS method was proposed. With the LVDI-UHPLC-MS/MS method, the sample can be directly injected into LC-MS analysis without any evaporation–reconstitution step, which is time-consuming and risks crucial chemical degradation during the evaporation procedure. Under the assistance of the injection solvent optimization, the validated LVDI-UHPLC-MS/MS-based method turned out to be extremely sensitive, accurate, and qualified for the bioassay measurement.

The pharmacokinetic results of HSYA, GRg_1_, GRe, GRb_1_, and NGR_1_ indicated that after combination, the absorption of these active components was increased inferred from their higher *C*_max_ and AUC_0-t_ values over that of the individual extract groups (Table 1). The increment of *C*_max_ and AUC_0-t_ values suggested that CYP450-mediated HHIs between CTE and NGTS may primarily account for the compatibility mechanisms of CTE and NGTS. An in vitro cocktail assay was then carried out to find the clues being responsible for HHIs between CTE and NGTS. The results showed CNP exhibited more potent inhibition on CYP1A2 (Table 2), the key enzyme involved in the oxidation reactions of most xenobiotics [20], compared with CTE and NGTS. In order to search for the single components contained in CNP responsible for the inhibition of CYP1A2 and other CYP450s, 18 main components from CNP were evaluated for their inhibition on CYP450s.

The results showed that GRg_1_ (A1), GRb_1_ (A2), GRd (A3), 6-hydoxykaempferol-3-*O*-glucoside (A6), kaempferol-3-*O*-glucoside (A7), anhydroxysafflor yellow B (AHSYB, A8), hydroxysafflor yellow A (HSYA, A9), 6-hydroxykaempferol-3,6-di-*O*-glucoside (A13), quercetin (A16), kaempferol (A17), and 6-hydroxykaempferol (A18) were the main active components for the CYP450 inhibition, and GRg_1_ (A1), GRb_1_ (A2), GRd (A3), quercetin (A16), kaempferol (A17), and 6-hydroxykaempferol (A18) were the main active components for CYP1A2 inhibition.

To further discuss the possibility of in vivo interaction between CTE and NGTS, the inhibitions of GRg_1_, GRb_1_, GRd, HSYA, NGR_1_, and GRe to CYP450s at their *C*_max_ levels were calculated by their respective “dose–response curve”. The results (Table S13) showed GRg_1_, GRb_1_, GRd, HSYA, NGR_1_, and GRe could not significantly inhibit CYP450s at their *C*_max_ levels, which is in accordance with the results that the combination use of CTE and NGTS can only increase the system exposures of these components to some extent.

## 4. Conclusions

In conclusion, the findings gained from the comparative pharmacokinetic investigations revealed there were pharmacokinetic interactions between CTE and NGTS, which may explain the integrative mechanisms of CNP and provide the experimental data and theoretical basis for further development and clinical applications of CNP. The developed LVDI-UHPLC-MS/MS method and pharmacokinetic interaction-based strategy provide a viable orientation for the compatibility investigation of herb medicines.

## Figures and Tables

**Table 1:** Pharmacokinetic parameters of hydroxysafflor yellow A (HSYA), ginsenoside Re (GRe), ginsenoside Rb1 (GRb_1_), ginsenoside Rd (GRd), ginsenoside Rg_1_ (GRg_1_), and notoginsenoside R_1_ (NGR_1_) after oral administration of CTE, NGTS, and CNP to rats. Each point represents the mean ± SD (*n* = 6).

Analyte	Group	*t*_1/2_ (h)	*T*_max_ (h)	*C*_max_ (ng·mL^−1^)	AUC_0–t_ (ng·h·mL^−1^)	AUC_0–∞_ (ng·h·mL^−1^)
HSYA	CTE	2.01 ± 0.34	0.88 ± 0.54	12.16 ± 3.09	33.05 ± 10.70	33.85 ± 10.78
	CNP	1.68 ± 0.79	1.17 ± 0.41	15.17 ± 4.39	38.33 ± 8.42	38.94 ± 8.60
NGR_1_	NGTS	10.53 ± 3.06	1.15 ± 1.06	0.64 ± 0.18	6.30 ± 2.41	8.14 ± 3.60
	CNP	12.36 ± 4.48	1.04 ± 0.97	0.79 ± 0.18	8.12 ± 1.53	11.49 ±3.54
GRb_1_	NGTS	36.89 ± 9.65	5.50 ± 1.80	113.08 ± 41.78	2317.66 ± 682.70	2808.87 ± 617.99
	CNP	34.47 ± 8.45	6.33 ± 3.45	129.00 ± 65.97	2472.33 ± 394.72	2816.01 ± 563.44
GRd	NGTS	42.75 ± 8.84 *	4.50 ± 1.76	29.43 ± 10.69	460.90 ± 117.22	618.90 ± 157.23
	CNP	27.79 ± 6.94	6.33 ± 3.44	31.07 ± 16.78	459.04 ± 51.04	549.18 ± 58.09
GRg_1_	NGTS	11.68 ± 2.09 *	4.71 ± 3.71	0.78 ± 0.13	13.99 ± 5.03	16.72 ± 3.93
	CNP	15.11 ± 8.89	5.38 ± 4.66	1.04 ± 0.34	16.69 ± 3.42	20.23 ± 3.35
GRe	NGTS	5.25 ± 2.27	2.60 ± 0.89*	0.29 ± 0.04*	1.82 ± 0.19	2.90 ± 0.60
	CNP	5.99 ± 3.86	0.80 ± 0.41	0.39 ± 0.08	1.93 ± 0.19	3.33 ± 1.13

*: *p* < 0.05, versus the combination group. *T*_max_: the time of peak concentration; t_1/2_: half-life; *C*_max_: the peak or maximum concentration; AUC: area under concentration-time curve. For abbreviations of analytes A6–A18, please refer to the Supplementary Materials section.

**Table 2:** IC_50_ values of CTE, NGTS, CNP, and the 18 representative compounds for inhibiting cytochrome p450 (CYP450) isozymes.

No.	IC_50_ (µg·mL^−1^/μM) ^#^						
	CYP2C9	CYP2E1	CYP2C19	CYP2D6	CYP2B6	CYP1A2	CYP3A4
CNP	21.66 ± 1.15	31.85 ± 4.15	62.95 ± 1.17	25.27 ± 0.25	52.19 ± 2.24	26.36 ± 6.98	91.77 ± 5.16
CTE	183.30 ± 17.98	35.47 ± 2.98	>200	126.17 ± 17.63	58.09 ± 13.41	138.60 ± 14.36	>200
NGTS	*9.32 ± 0.56*	16.12 ± 2.59	40.85 ± 6.98	*12.23 ± 4.25*	43.90 ± 7.91	60.68 ± 2.26	106.27 ± 14.00
GRg_1_ (A1)	36.49 ± 1.32	34.08 ± 4.76	78.12 ± 5.25	55.73 ± 6.08	*11.02 ± 0.87*	*13.09 ± 8.24*	39.25 ± 4.57
GRb_1_ (A2)	17.57 ± 2.03	27.96 ± 2.39	16.80 ± 2.52	17.06 ± 1.29	120.83 ± 10.89	*5.84 ± 0.14*	30.04 ± 2.47
GRd (A3)	73.01 ± 3.71	43.46 ± 0.06	71.36 ± 14.28	64.81 ± 11.68	>200	*4.36 ± 2.31*	61.43 ± 14.32
GRe (A4)	>200	25.59 ± 9.03	50.53 ± 15.39	>200	39.40 ± 4.81	94.41 ± 4.52	75.11 ± 7.24
NGR_1_ (A5)	154.47 ± 16.65	121.70 ± 8.96	107.59 ± 29.58	20.42 ± 3.45	>200	112.07 ± 7.72	84.59 ± 1.79
A6	55.84 ± 3.40	40.76 ± 1.65	53.67 ± 0.22	19.46 ± 2.83	114.70 ± 3.04	52.77 ± 1.93	26.45 ± 1.39
A7	80.47 ± 1.05	*13.59 ± 0.60*	18.50 ± 1.60	31.02 ± 4.17	177.17 ± 4.21	153.27 ± 8.73	126.70 ± 4.24
A8	*1.21 ± 0.51*	*9.06 ± 1.85*	111.26 ± 21.57	*10.54 ± 1.11*	>200	>200	34.38 ± 9.16
A9 (HSYA)	*0.17 ± 0.02*	*0.73 ± 0.15*	49.33 ± 3.28	*0.14 ± 0.06*	>200	>200	*9.42 ± 2.26*
A10	39.06 ± 0.09	>200	>200	>200	>200	>200	>200
A11	24.92 ± 4.96	*5.45 ± 1.37*	>200	64.55 ± 7.71	17.33 ± 0.96	53.17 ± 6.12	16.18 ± 4.30
A12	>200	>200	>200	98.56 ± 12.30	>200	>200	>200
A13	93.00 ± 2.56	*10.22 ± 0.65*	48.91 ± 4.36	37.12 ± 8.85	21.02 ± 2.31	*13.35 ± 2.27*	58.52 ± 0.66
A14	>200	>200	>200	>200	>200	>200	>200
A15	>200	>200	>200	>200	>200	127.47 ± 6.55	>200
A16	*13.48 ± 2.41*	20.29 ± 4.13	46.35 ± 7.05	31.11 ± 3.91	68.48 ± 9.88	41.22 ± 3.18	73.74 ± 13.64
A17	*14.92 ± 4.24*	32.46 ± 5.79	70.38 ± 1.18	59.17 ± 1.01	>200	21.46 ± 4.50	117.00 ± 6.77
A18	*2.13 ± 0.64*	*4.27 ± 1.30*	*1.79 ± 0.52*	*0.98 ± 0.38*	*4.42 ± 1.31*	*0.12 ± 0.01*	*2.48 ± 0.48*

^#^: Values were obtained from triplicate tests, and presented as mean ± SD. The units of IC_50_ values for CTE, NGTS, and CNP are µg·mL^−1^, whereas for the 18 single components are μM. Potent inhibitors (IC_50_ < 15 μM for 1A2, 2C9, 2C19, 2D6, 2B6, and 3A4) are presented in italic. For abbreviations of analytes A6–A18, please refer to the Supplementary Materials section.

## Supplementary Materials

The following are available online at https://www.mdpi.com/1999-4923/12/2/180/s1: Figure S1. The total ion current chromatogram (TIC) of CTE; the corresponding chemical composition information was reported in previous research (Chen, et al., 2014; Analyst 139, 6474–6485). Figure S2. The optimization of sample solvents for the pharmacokinetic analysis (A) and the cocktail assay (B) (*n* = 3). Figure S3. Kinetic profiles for the enzymatic turnover of CYP450-mediated probe reactions. Figure S4. Inhibition curves of the seven positive inhibitors obtained from the substrate cocktail incubation. Table S1. Multiple reaction monitoring transitions and fragmentation parameters of six standards and IS1 for PK analysis. Table S2. Multiple reaction monitoring transitions and fragmentation parameters of seven metabolites and two internal standards (IS2 and IS3) for cocktail assay. Table S3. The instrument stability of the LVDI-UHPLC-MS/MS setup. Table S4. Regression equations, linear ranges, and low limits of quantification (LLOQ) of the six standards in rat plasma for the PK study. Table S5. Intra- and inter-day precisions and determination accuracies of six standards for the pharmacokinetic study. Table S6. Extract recoveries and matrix effects of six target constituents in rat plasma samples for the PK study. Table S7. Stability of the six CNP constituents in rat plasma samples for the PK study. Table S8. Plasma concentration time of the six target constituents after oral administration of CTE, NGTS, and CNP. Table S9. Regression equations, linear ranges, and LLOQs of the seven metabolites for the cocktail analysis. Table S10. Intra- and inter-day precisions and determination accuracies of the seven metabolites for cocktail analysis. Table S11. Extract recoveries and matrix effects of seven target constituents and two ISs for the cocktail analysis. Table S12. Michaelis constant (Km) determined for the enzymatic reaction of the probe substrates and the inhibition IC_50_ values were measured for the positive inhibitors of seven CYP450s. Table S13. Responses (% control) of HSYA, GRb_1_, GRd, GRe, GRg_1_, and NGR_1_ at their *C*_max_ levels in the rat plasma.
