# Supplementary material for: Large Volume Direct Injection Ultra-High Performance Liquid Chromatography–Tandem Mass Spectrometry-Based Comparative Pharmacokinetic Study between Single and Combinatory Uses of Carthamus tinctorius Extract and Notoginseng Total Saponins
Source: Pharmaceutics. 2020 Feb 20;12(2):180. doi: 10.3390/pharmaceutics12020180 (PMC7076352; doi:10.3390/pharmaceutics12020180)
Supplement: Supplementary file 1 [file pharmaceutics-12-00180-s001.pdf]

pharmaceutics

MDPI

Supplementary Materials: Large Volume Direct Injection-Ultra High Performance Liquid Chromatography-Tandem Mass Spectrometry-Based Comparative Pharmacokinetic Study between Single and Combinatory Uses of Carthamus Tinctorius Extract and Notoginseng Total Saponins

Jinfeng Chen, Xiaoyu Guo, Yingyuan Lu, Mengling Shi, Haidong Mu, Yi Qian, Jinlong Wang, Mengqiu Lu, Mingbo Zhao, Pengfei Tu, Yuelin Song, Yong Jiang

CONTENTS

1. Materials and methods
2. Results
3. Supplementary tables and figures

Figure S1. The total ion current chromatogram (TIC) of CTE, the corresponding chemical composition information were reported on the previous researches (Chen, et al., 2014; Analyst 139, 6474–6485).
Figure S2. The optimization of sample solvents for the pharmacokinetic analysis (A) and the cocktail assay (B) ($n = 3$).
Figure S3. Kinetic profiles for the enzymatic turnover of CYP450-mediated probe reactions.
Figure S4. Inhibition curves of the seven positive inhibitors obtained from the substrate cocktail incubation.
Table S1 Multiple reaction monitoring transitions and fragmentation parameters of six standards and IS1 for PK analysis.
Table S2 Multiple reaction monitoring transitions and fragmentation parameters of seven metabolites and two internal standards (IS2 and IS3) for cocktail assay.
Table S3 The instrument stability of the LVDI-UHPLC-MS/MS setup.
Table S4 Regression equations, linear ranges, and low limits of quantification (LLOQ) of the six standards in rat plasma for the PK study.
Table S5 Intra- and inter-day precisions and determination accuracies of six standards for the pharmacokinetic study.
Table S6 Extract recoveries and matrix effects of six target constituents in rat plasma samples for the PK study.
Table S7 Stability of the six CNP constituents in rat plasma samples for the PK study
Table S8 Plasma concentration-time of the six target constituents after oral administration of CTE, NGTS, and CNP, respectively.
Table S9 Regression equations, linear ranges, LLOQs of the seven metabolites for the cocktail analysis.
Table S10 Intra- and inter-day precisions and determination accuracies of the seven metabolites for cocktail analysis.
Table S11 Extract recoveries and matrix effects of seven target constituents and two IS for the cocktail analysis.
Table S12 *Km* values determined for the enzymatic reaction of the probe substrates and the inhibition IC_{50} values measured for the positive inhibitors to seven CYP450s.

Table S13 Responses (% control) of HSYA, GRb_1, GRd, GRe, GRg_1, and NGR_1 at their C_{max} levels in the rat plasma.

1. Materials and methods

1.1 Chemicals and reagents

Ginsenosides Rg_1 (GRg_1, A1), Rb_1 (GRb_1, A2), Rd (GRd, A3), Re (GRe, A4), notoginsenoside R_1 (NGR_1, A5), hydroxysafflor yellow A (HSYA, A9), quercetin (A16), kaempferol (A17), and linarin (IS1) were purchased from Chengdu Must Bio-Tech Co., Ltd (Chengdu, China). 6-Hydoxykaempferol-3-*O*-glucoside (A6), kaempferol-3-*O*-glucoside (A7), anhydroxysafflor yellow B (AHSYB, A8), 6-hydroxykaempferol-3,6,7-*tri*-*O*-glucoside (A10), kaempferol-3-*O*-rutinoside (A11), 6-hydroxykaempferol-3-*O*-rutinosyl-6-*O*-glucoside (A12), 6-hydroxykaempferol-3,6- *di*-*O*-glucoside (A13), kaempferol-3-*O*-sophoroside (A14) and 6-hydroxyapigenin-6-*O*-glucosyl-7-*O*-glucuronide (A15) were previously isolated from *Carthamus tinctorius* extract (CTE), and their structures were identified *via* analysis of their spectroscopic data (UV, MS and NMR) [1]. 6-Hydoxykaempferol (A18) was purchased from Biopeony Beijing Co., Ltd. Nicotinamide adenine dinucleotide phosphate hydrate (NADPH), paracetamol (dEtPHE), midazolam (MID), tolbutamide (TOL), dextromethorphan (DEX), chlorzoxazone (CHL), phenacetin (PHE), bupropion (BUP), triethylenethiophosphoramide (TRI), sulfaphenazole (SUL), ticlopidine (TIC), furafylline (FUR), ketoconazole (KET), quinidine (QUI), 4-methylpyrazole (MET), and dimethylsulfoxide (DMSO) were purchased from Sigma-Aldrich (St. Louis, MO). 1′-Hydroxymidazolam (OHMID), 6-hydroxychlorzoxazone (OHCHL), hydroxybupropion (OHBUP), hydroxytolbutamide (OHTOL), dextrorphan-D-tartrate (dMeDEX), 4′-hydroxydiclofenac-[^{13}C6] (OHDIC-[^{13}C6], IS2), and hydroxybupropion-[D6] (OHBUP-[D6], IS3) were purchased from BD Biosciences, Franklin Lakes, NJ, USA. 5-Hydroxyomeprazole (OHOME) was obtained from Toronto Research Chemicals Inc. (Toronto, Canada). Purities of all authentic compounds were determined to be greater than 98% by UHPLC-MS/MS.

Phosphate buffer salt solution (PBS, pH = 7.4) was supplied by Beijing Leagene Biotechnology Co., Ltd. Acetonitrile, methanol, and formic acid of optima® LC/MS grade were purchased from Thermo-Fisher (Rockford, IL, USA). Deionized water was prepared in-house using a Milli-Q (MQ) Integral Water Purification System (Millipore, Bedford, MA, USA). The other chemicals were of analytical grade and obtained commercially from Beijing Chemical Works (Beijing, China).

1.2 Plasma pretreatment for pharmacokinetic studies

Oasis® PRiME HLB SPE cartridges (1 cc/30 mg, Waters, Milford, MA), which were successively preconditioned with 5 mL of methanol and 5 mL of 0.05 M phosphoric acid aqueous solution, were used to process the plasma samples: 190 μL plasma samples were mixed with 10 μL formic acid (5%, *v*/*v*) and 10 μL IS1 (0.5 μg/mL), vortexed for 1 min, and centrifuged (12 000 rpm) for 10 min at 4 °C. The supernatant was diluted with 0.05 M phosphoric acid aqueous solution (1:1, *v*/*v*) and subsequently loaded onto a HLB column. Gradient elution was performed using 2 mL (500 μL × 4) of 0.05 M phosphoric acid aqueous solution, 1 mL (500 μL × 2) of 0.05 M phosphoric acid aqueous containing 5% methanol, 0.8 mL (400 μL × 2) methanol containing 2% formic acid, 0.8 mL (400 μL × 2) methanol, and 0.2 mL methanol containing 10 mM ammonium formate. All the methanol eluates were pooled and centrifuged at 12000 rpm for 10 min for two twice. Then an aliquot of 100 μL supernatant was diluted with 100 μL MQ-water and centrifuged at 12000 rpm for 10 min, before subjected to LVDI-UHPLC-MS/MS analysis.

1.3 Chromatography programs of LVDI-UHPLC-MS/MS for pharmacokinetic and cocktail studies

Both pharmacokinetic and cocktail studies chose an Acquity UPLC® HSS T3 column (50 mm × 2.1 mm i.d., particle size 1.8 μm, Waters, Ltd., USA), being protected by a Van Guard™ HSS T3 (5 mm × 2.1 mm i. d., 1.8 μm, Waters, USA) for chromatographic separations. Besides, their mobile phase consisted of 0.01% aqueous formic acid (A) and acetonitrile containing 0.01%

formic acid (B). For the loading phase of the pharmacokinetic analysis, the pumps were responsible for delivering 100%A at a high flow rate of 3 mL·min^{-1}, during 0.5 min. At its elution phase, the gradient with a total flow rate of 0.3 mL·min^{-1} was as follows: 0 – 6 min, 0% – 5% B; 6 – 7 min, 5% – 33% B; 7 – 7.5 min, 33% – 29% B; 7.5 – 9 min, 29% – 100% B; 9 – 12 min, 100% – 100% B. The column oven was maintained at 25 °C. For the cocktail assay, at the loading phase, the pumps were responsible for delivering 100%A to the pre-guard column at a flow rate of 0.4 mL·min^{-1}, during 0.5 min. At the elution phase, the gradient at a total flow rate of 0.2 mL·min^{-1} as follows: 0 – 5 min, 0% B; 5 – 7 min, 0% – 2% B; 7 – 8 min, 2% – 20% B; 8 – 13 min, 20% – 80% B; 13 – 15 min, 100% B. The column was maintained at 25 °C in the column oven. At the end of each run, the whole system was switched to the initial status and maintained for five minutes to re-equilibrate the system.

1.4 Mass spectrometric parameter optimization for pharmacokinetic study and in vitro cocktail assay

Stock solution of each reference standard was diluted to appropriate concentration (100 – 200 ng·mL^{-1}) with 50% aqueous methanol and directly infused (flow rate, 10 μL·min^{-1}) into the ion source of a QTRAP-MS *via* a syringe pump for mass parameter optimization. For the pharmacokinetic analysis, seven analysts, including HSYA, GRb$_1$, GRg$_1$, GRd, NGR$_1$, GRe and linarin (IS1) were involved as the targeted components. Negative polarity could afford better mass responses for those components in comparison with the positive ionization mode. Regarding the seven metabolites (dEtPHE, OHMID, OHTOL, dMeDEX, OHCHL, OHBUP, and OHOME) of the cocktail analysis, the optimum mass parameters were also obtained by manual tuning *via* directly infusing pure compounds into mass spectrometer; both positive and negative polarities were applied according to the results. Quantitative analyses were monitored in MRM mode. Mass axis was calibrated using standard polypropylene glycol (PPG) dilution solvents. The ion-spray voltages were maintained at –4500 V and 5500 V for the negative and positive polarities, respectively. Nitrogen was used as the nebulizer (GS1), curtain (CUR), heater (GS2), and collision gases. While the GS1, GS2, and CUR for the PK study were set as 45, 45, and 35 psi, respectively. GS1, GS2, and CUR for the cocktail assay were set as 50, 50, and 35 psi, respectively. The ion sources of PK and cocktail studies were separately heated to 450 °C and 500 °C. The precursor-to-product transition, optimized declustering potential (DP) values, and collision energy (CE) values of the PK and cocktail studies are separately shown in Table S1 and Table S2, whereas the dwell time, entrance potential (EP), and collision cell exit potential (CXP) values of all ion transitions were fixed at 30 ms, 10 V, and 12 V, respectively.

The injection volume of PK study was set as 100 μL (50 μL sample for two times by LVDI). The preparation and measurement of the drug-free samples were performed in parallel with those of the treated samples. For the cocktail study, the injection volume was set as 50 μL. The preparation and measurement of the drug-free samples were performed in parallel with those of the treated samples.

1.5 Method validations

Mixed standard stock solutions were individually obtained by pooling all stock solutions (HSYA/GRg$_1$/GRb$_1$/GRd/GRe/NGR$_1$ for the PK study, dEtPHE/OHOME /OHTOL/dMeDEX/OHMID/OHBUP/OHCHL for the cocktail assay), and the obtained solutions were then sequentially diluted using 50% aqueous methanol to afford serial mixed standard solutions with desired concentration levels. Four concentration levels of calibration samples, including high, medium (two concentration levels), and low levels, were selected as quality control (QC) samples. The method validation, in terms of selectivity, linearity and sensitivity, precision and accuracy, recovery and matrix, and stability, was conducted following the US Food and Drug Administration (FDA) Guidance on Bioanalytical Method Validation and Drug Interaction studies [2,3].

2. Results

2.1 The instrument precision of the LVDI-UHPLC-MS/MS

It is well known the instrument stability is very important for establishing a quantitative

method. Contrary to the common UHPLC-MS methods, the setup of LVDI-UHPLC-MS/MS was installed using additional pipelines to connect the UHPLC, the 6-port/2-channel switching valve, and the QTRAP-MS. Therefore, the stability of LVDI-UHPLC-MS/MS setup was firstly tested and verified by injecting 5 μL HQC and LQC samples before optimizing the chromatographic programs of the loading phases. The results (Table S3) indicated that the instrument stability of the LVDI-UHPLC-MS/MS setup could meet the demands for developing a quantitative method.

2.2 Optimization the elution phase program of LVDI-UHPLC-MS/MS

The elution programs of the LVDI-UHPLC-MS/MS for the PK and cocktail studies were separately optimized. Because of the pivotal role for the chromatographic performances, the analytical columns were carefully screened. For the PK study, the HSS T3 column (50 × 2.1 mm, I.D, 1.8 μm) was advantageous at resolution, peak shape, and chromatographic retention of HSYA in comparison with BEH C_{18} (50 × 2.1 mm, I.D, 1.7 μm) and RP shield C_{18} (50 × 2.1 mm, I.D, 1.7 μm) columns. In the cocktail assay, the T3 column showed more strong retention of dEtPHE than the BEH C_{18} column, and obtained better peak shapes of OHBUP and OHMID than the RP shield C_{18} column. Thus, both PK and cocktail studies employed HSS T3 column based on the resolution, peak shape, and chromatographic retention. Regarding the HSS T3 column, the gradient water and acetonitrile were employed as elution solvents after careful assessments between water and acetonitrile and water and methanol. Both PK and cocktail studies introduced formic acid (0.01%, *v*/*v*) as the solvent additive since it could afford better peak shapes along with overall MRM responses than ammonium formate (1, 5, 10 mM). In total, the ammonium formate additives can induce peak shape distortions of HSYA, dEtPHE, OHBUP, and OHMID. Afterwards, the gradient programs of the elution phases were individually customized to afford satisfactory chromatographic separations for the pharmacokinetic and cocktail studies. Furthermore, a relative lower temperature (25 °C) was applied for the analytical column, which could significantly modify the peak shapes of these analytes, especially for the HSYA and dEtPHE, in comparison with those higher temperatures, *e.g.* 40 °C and 50 °C. Consequently, the gradient programs of the loading phases were optimized based on the above chromatography programs of the elution phases.

2.3 Results of method validation

2.3.1 Specificity

For the PK study, representative MRM chromatograms obtained from blank rat plasma, a blank plasma sample spiked with six analytes and an internal standard (IS1), and the plasma sample after oral administration of CNP were respectively shown in Figure 2. For the cocktail assay, representative MRM chromatograms obtained from the incubation matrix, an incubation matrix spiked with seven metabolites, and two internal standards (IS2 and IS3), were respectively shown in Figure 3. No significant interferences of endogenous ingredients were observed for the LVDI-UHPLC-MS-based methods for the PK and cocktail studies.

2.3.2 Linearity and Sensitivity

Linear regression equations for calibration curves of the six standards for the PK study and the seven metabolites for the cocktail assay were respectively summarized in Table S4 and Table S9. The calibration curves covered a wide dynamic range and the correlation coefficients of all constituents were more than 0.9911 in the linear range.

2.3.3 Precision and Accuracy

As shown in Table S5 and Table S10, RSDs of intra- and inter-day precisions were found to be lower than 15.15% for the PK and cocktail studies. The accuracy of the PK and cocktail studies were respectively in the ranges of 88.21–104.79% and 86.52–107.60% at four-level QC samples. All the assay values satisfied the acceptable criteria, indicating the favorable data for precision and accuracy of this developed LVDI-UHPLC-MS/MS method.

2.3.4 Extraction Recovery and Matrix Effect

Matrix effects and extraction efficiency were examined in duplicate by three groups of

standard addition experiments. Each group included four concentration levels. For the PK study, the extraction efficiencies of HSYA, GRg_1, NR_1, and GRe ranged from 90.23% to 110.26% at all the four concentrations (Table S6). Their matrix effect led to weak ion suppression, ranging from −2.4% to 15.4% for all the four concentrations (Table S6). Considering the higher plasma concentrations of Rb_1 and Rd, their extraction efficiency and matrix effects were compromised (around 70%) to improve the sensitivity of the other four analytes.

The extraction recoveries of the seven metabolites for the cocktail assay at four concentration levels ranged from 82.06% to 114.70% (Table S11), indicating the recovery of protein precipitation with methanol was precious and proper for various levels. And the matrix effects were in the range of 83.03% to 114.17% at four QC levels (Table S11). Therefore, there were no obvious matrix effects for the analysis of target compounds and two internal standards (IS2, IS3) in the cocktail investigation, showing that the endogenous ingredients did not interfere with the ionization of the target analytes.

2.3.5 Stability

The stabilities of the six target constituents in the rat plasma samples were listed in Table S7. The results showed that these constituents in plasma were all stable in autosampler at 4 °C for 24 h, at −80 °C for 60 days, and three freeze/thaw cycles, with RSD values in the range of 0.22% to 16.34%.

Above all, the newly developed methods based on LVDI-UHPLC-MS/MS were sensitive, precise, and accurate for the pharmacokinetic and cocktail assays.

2.3.6 Optimization and verification of the incubation system for the cocktail assay

The probe compounds, *viz*. PHE, OME, TOL, DEX, MID, CHL, and BUP were finally chosen after incubating all the recruited substrates. In order to assure the linear relationship between enzyme activity and metabolic transformation, the protein concentration should be in the range of 0.05–0.20 mg/mL, and the incubation time should be among 0–20 min. Therefore, the incubation was conducted using 0.20 mg/mL protein for 15 min. The incubation system was also optimized in terms of substrate concentrations. The substrates concentrations of PHE/OME/TOL/DEX /MID/CHL/BUP were respectively set at 90/1.07/18/0.13/0.02/3.6/90 μM with the assistance of LVDI-UHPLC-MS/MS, which were lower than the most reports [4–13]. The *Km* and IC_{50} values for known CYP450 substrates and inhibitors are shown in Figures S3 & S4, and Table S12. The measured values were in good agreement with recently published literature [5–13], demonstrating the applicability of the assay.

References

[1] Chen, J.; Tu, P.; Jiang, Y. HPLC fingerprint-oriented preparative separation of major flavonoids from safflower extract by preparative pressurized liquid chromatography. *J. Chin. Pharma. Sci.* **2014**, 23, 6; DOI: 10.5246/jcps.2014.07.064.

[1] FDA. U.S. Food and Drug Administration. Bioanalytical Method Validation Guidance for Industry. 2018. http://www.fda.gov/downloads/Drugs/Guidance-Compliance RegulatoryInformation/Guidances/UCM070107.pdf.

[2] FDA. U.S. Food and Drug Administration. Clinical Drug Interaction Studies-Study Design, Data Analysis, and Clinical Implications Guidance for Industry. 2017. http://www.fda.gov/downloads/Drugs/GuidanceComplianceRegulatoryInformation/Guidances/UCM072101.pdf. .

[3] Walsky R.L.; Obach R.S. Validated assays for human cytochrome P450 activities. *Drug Metab. Dispos.* **2004**, 32, 647-660; DOI: 10.1124/dmd.32.6.647.

[4] Murray J.; Picking D.; Lamm A.; McKenzie J.; Hartley S.; Watson C.; Williams L.; Lowe H.; Delgoda R. Significant inhibitory impact of dibenzyl trisulfide and extracts of Petiveria alliacea on the activities of major drug-metabolizing enzymes in vitro: An assessment of the potential for medicinal plant-drug interactions. *Fitoterapia* **2016**, 111, 138-146; DOI: 10.1016/j.fitote.2016.04.011.

[5] Testino S.A., Jr., Patonay G. High-throughput inhibition screening of major human cytochrome P450 enzymes using an in vitro cocktail and liquid chromatography-tandem mass spectrometry. *J. Pharm. Biomed. Anal.* **2003**, 30, 1459-1467; DOI: 10.1016/s0731-7085(02)00480-6.

[6] Turpeinen M.; Nieminen R.; Juntunen T.; Taavitsainen P.; Raunio H.; Pelkonen O. Selective inhibition of CYP2B6-catalyzed bupropion hydroxylation in human liver microsomes in vitro. *Drug Metab. Dispos.* **2004**, 32, 626-631; DOI: 10.1124/dmd.32.6.626.

[7] Kozakai K.; Yamada Y.; Oshikata M.; Kawase T.; Suzuki E.; Haramaki Y.; Taniguchi H. Reliable high-throughput method for inhibition assay of 8 cytochrome P450 isoforms using cocktail of probe substrates and stable isotope-labeled internal standards. *Drug Metab. Pharmacokinet* **2012**, 27, 520-529; DOI: 10.2133/dmpk.dmpk-12-rg-014.

[8] He F.; Bi H.C.; Xie Z.Y.; Zuo Z.; Li J.K.; Li X.; Zhao L.Z.; Chen X.; Huang M. Rapid determination of six metabolites from multiple cytochrome P450 probe substrates in human liver microsome by liquid chromatography/mass spectrometry: application to high-throughput inhibition screening of terpenoids. *Rapid Commun. Mass Spectrom.* **2007**, 21, 635-643; DOI: 10.1002/rcm.2881.

[9] Qiu F; Zhang R; Sun J; Jiye A; Hao H; Peng Y; Ai H; Wang G. Inhibitory effects of seven components of danshen extract on catalytic activity of cytochrome P450 enzyme in human liver microsomes. *Drug Metab. Dispos.* **2008**, 36, 1308-1314; DOI: 10.1124/dmd.108.021030.

[10] Bu HZ; Knuth K; Magis L; Teitelbaum P. High-throughput cytochrome P450 inhibition screening via cassette probe-dosing strategy. IV. Validation of a direct injection on-line guard cartridge extraction/tandem mass spectrometry method for simultaneous CYP3A4, 2D6 and 2E1 inhibition assessment. *Rapid Commun. Mass Spectrom.* **2000**, 14, 1943-1948; DOI: 10.1002/1097-0231(20001030)14:20<19 43:AID-RCM116>3.0.CO;2-F.

[11] Dierks E.A.; Stams K.R.; Lim H.K.; Cornelius G.; Zhang H.; Ball S.E. A method for the simultaneous evaluation of the activities of seven major human drug-metabolizing cytochrome P450s using an in vitro cocktail of probe substrates and fast gradient liquid chromatography tandem mass spectrometry. *Drug Metab. Dispos.* **2001**, 29, 23-29;

Table S1: Multiple reaction monitoring transitions and fragmentation parameters of six standards and internal standard for the PK analysis.

Compound	Time (ms)	Q1 (Da)	Q3 (Da)	DP (V)	CE (eV)
HSYA	50	611.1	491.0	–150	–36
GRg_1	50	845.5	799.5	–85	–38
GRb_1	50	1153.5	1107.4	–135	–37
NGR_1	50	977.5	931.5	–98	–30
GRd	50	991.5	945.5	–130	–37
GRe	50	991.5	945.5	–130	–37
Linarin, IS1	50	591.2	283.1	–120	–58

DP: declustering potential; CE: collision energy.

For abbreviations of analytes please refer to the "Chemicals and reagents" section.

Table S2: Multiple reaction monitoring transitions and fragmentation parameters of seven metabolites and two internal standards for the cocktail assay.

Compound	Time (min)	Ion mode	Q1 (Da)	Q3 (Da)	DP (V)	CE (eV)
dMeDEX	11.0	pos	258.1	157.1	60	52
OHMID	11.8	pos	342.2	203.3	100	36
dEtPHE	10.8	pos	152.1	110.0	95	23
OHBUP	11.1	pos	256.1	238.1	100	17
OHOME	11.2	pos	362.2	214.1	80	10
OHBUP-[D_6], IS1	11.1	pos	262.1	244.1	95	23
OHCHL	11.4	neg	183.7	119.9	–70	–25
OHTOL	11.9	neg	285.0	186.1	–88	–28
OHDIC-[$^{13}C_6$], IS2	13.3	neg	318.2	274.1	–50	–20

DP: declustering potential; CE: collision energy.

For abbreviations of analytes please refer to the "Chemicals and reagents" section.

Table S3: The instrument stability of the LVDI-UHPLC-MS/MS setup (n = 6).

**Analyte**	**High concentration**			**Low concentration**		
	**Spiked (ng·mL^{-1})**	**Peak area (mean ± SD)**	**RSD (%)**	**Spiked (ng·mL^{-1})**	**Peak area (mean ± SD)**	**RSD (%)**
HSYA	30	79400±1345	1.70	6	7690±278	3.61
NGR$_1$	20.8	62600±820	1.31	4.16	9660±424	4.39
GRb$_1$	20.5	12300±1173	9.53	4.1	2018±290	14.37
GRd	26.4	122000±6285	5.15	5.28	14800±689	4.65
GRe	33	474000±21159	4.46	6.6	56900±4251	7.47
GRg$_1$	20.8	66200±1859	2.81	4.16	14500±443	3.06

For abbreviations of analytes please refer to the "Chemicals and reagents" section.

Table S4: Regression equations, linear ranges, low limits of quantification (LLOQs) and low limits of quantification (LLODs) of six standards in rat plasma for the PK study.

Analyte	Regression equation	r	Linear range (ng·mL^{-1})	LLOQ (ng·mL^{-1})	LLOD (ng·mL^{-1})
HSYA	$y = 0.746\ x + 0.0527$	0.9927	0.083 – 16.67	0.06	0.02
GRg$_1$	$y = 3.18\ x - 0.108$	0.9914	0.058 – 11.56	0.06	0.03
NGR$_1$	$y = 5.68\ x + 0.053$	0.9929	0.058 – 11.56	0.02	0.01
GRd	$y = 1.1\ x - 3.76$	0.9911	2.28 – 183.00	0.76	0.24
GRb$_1$	$y = 0.573\ x - 1.14$	0.9915	5.69 – 456.00	1.89	0.63
GRe	$y = 5.62\ x - 0.32$	0.9919	0.092 – 18.33	0.06	0.03

For abbreviations of analytes please refer to the "Chemicals and reagents" section.

Table S5: Intra- and inter-day precisions and determination accuracies of six standards for the pharmacokinetic study.

Analyte	Spiked (ng·mL^{-1})	Intra-day (mean ± SD, n = 4)			Inter-day (mean ± SD, n = 6)		
		Measured (ng·mL^{-1})	Accuracy (%)	Precision (RSD, %)	Measured (ng·mL^{-1})	Accuracy (%)	Precision (RSD, %)
HSYA	0.092	0.089±0.008	96.74	8.99	0.095±0.010	103.26	10.52
	0.417	0.397±0.031	95.20	7.81	0.427±0.050	102.40	11.71
	1.67	1.75±0.25	104.79	14.29	1.65±0.19	98.80	11.52

	16.70	15.74±1.08	94.25	6.86	16.77±2.06	100.42	12.28
NGR$_1$	0.058	0.060±0.002	103.45	3.33	0.056±0.005	96.55	8.93
	0.144	0.135±0.016	93.75	11.85	0.136±0.017	94.44	12.50
	0.578	0.602±0.061	104.15	10.13	0.558±0.055	96.54	9.86
	5.780	6.050±0.327	104.67	5.40	5.530±0.539	95.67	9.75
GRb$_1$	2.280	2.298±0.026	100.79	1.13	2.345±0.061	102.85	2.60
	5.690	5.630±0.095	98.95	1.69	5.450±0.414	95.78	7.60
	22.80	21.85±1.56	95.83	7.14	22.84±1.27	100.18	5.56
	228.0	234.0±11.6	102.63	4.96	226.6±7.4	99.39	3.27
GRd	0.917	0.916±0.010	99.89	1.09	0.920±0.005	100.33	0.54
	2.29	2.27±0.05	99.13	2.20	2.33±0.02	101.75	0.86
	9.17	8.24±0.73	89.86	8.86	8.09±0.70	88.22	8.65
	91.7	90.78±6.53	99.00	7.19	86.08±9.77	93.87	11.35
GRg$_1$	0.058	0.059±0.003	101.72	5.08	0.058±0.005	100.00	8.62
	0.144	0.138±0.010	95.83	7.25	0.151±0.007	104.86	4.64
	0.578	0.599±0.028	103.63	4.67	0.552±0.066	95.50	11.96
	5.78	5.78±0.57	100.00	9.86	6.03±0.63	104.33	10.45
GRe	0.0917	0.0917±0.0007	100.00	0.76	0.0938±0.0004	102.29	0.43
	0.229	0.228±0.006	99.56	2.63	0.228±0.018	99.56	7.89
	0.917	0.947±0.007	103.27	0.74	0.906±0.101	98.80	11.15
	9.17	9.47±1.08	103.27	11.40	9.23±1.14	100.65	12.35

For abbreviations of analytes please refer to the "Chemicals and reagents" section.

Table S6: Extraction recoveries and matrix effects of six target constituents in rat plasma samples (n = 3, mean ± SD).

Analyte	Spiked (ng·mL^{-1})	Recovery (%)	RSD (%)	Matrix effect (%)	RSD (%)
HSYA	0.092	107.73±3.70	3.43	92.77±17.71	19.09
	0.417	101.76±4.38	4.30	100.85±12.69	12.58
	1.67	110.26±5.45	4.94	99.24±10.79	10.87
	16.70	96.05±5.62	5.85	97.04±10.50	10.82
NGR$_1$	0.058	99.99±10.40	10.40	98.56±8.81	8.94
	0.144	96.08±3.31	3.45	92.29±8.12	8.80
	0.578	92.94±8.48	9.12	89.36±2.73	3.06
	5.78	98.30±11.64	11.84	87.81±1.67	1.90
GRb$_1$	2.28	71.26±5.56	7.80	71.40±1.58	2.21
	5.69	58.51±5.43	9.28	65.29±7.63	11.69

	22.80	61.30±6.57	10.72	57.50±2.45	4.26
	228	66.62±7.52	11.29	68.36±5.61	8.21
GRd	0.917	65.65±6.46	9.84	63.43±10.06	15.86
	2.29	67.04±9.30	13.87	77.99±9.18	11.77
	9.17	69.05±8.03	11.63	78.17±2.56	3.27
	91.70	63.19±3.92	6.20	56.61±1.21	2.14
GRg_1	0.058	94.98±13.09	13.78	99.02±6.59	6.66
	0.144	100.39±3.65	3.64	94.32±6.26	6.64
	0.578	90.74±9.04	9.96	98.62±3.04	3.08
	5.78	95.32±2.58	2.71	88.46±3.26	3.69
GRe	0.0917	106.66±6.90	6.47	99.79±15.45	15.48
	0.229	101.06±1.83	1.81	94.51±3.13	3.31
	0.917	90.23±5.60	6.21	97.31±1.89	1.94
	9.17	105.98±8.80	8.30	95.52±4.99	5.22
IS1 (linarin)	2.78	92.77±6.89	7.43	93.83±8.08	8.61

For abbreviations of six standards please refer to the “Chemicals and reagents” section.

Table S7: Stability of the six CNP constituents in rat plasma samples (mean ± SD, *n* = 3).

Analyte	Spiked ($ng \cdot mL^{-1}$)	Stored at room temperature for 24 h			Three freeze-thaw cycles			Stored at ⊚80 °C for 60 days		
		Measured ($ng \cdot mL^{-1}$)	Accuracy (%)	RSD (%)	Measured ($ng \cdot mL^{-1}$)	Accuracy (%)	RSD (%)	Measured ($ng \cdot mL^{-1}$)	Accuracy (%)	RSD (%)
HSYA	0.167	0.172±0.013	102.99	7.56	0.163±0.016	97.60	9.82	0.167±0.004	100.00	2.40
	0.417	0.392±0.032	94.00	8.16	0.424±0.014	101.68	3.30	0.412±0.033	98.80	8.01
	1.67	1.82±0.12	108.98	6.59	1.84±0.14	110.18	7.61	1.58±0.07	94.61	4.43
	16.70	15.60±0.50	93.41	3.21	15.20±0.80	91.02	5.26	16.60±1.10	99.40	6.63
NGR_1	0.0578	0.0527±0.0002	91.18	0.38	0.0528±0.0004	91.35	0.76	0.0593±0.0064	102.60	10.79
	0.289	0.320±0.019	110.73	5.94	0.327±0.013	113.15	3.98	0.281±0.016	97.23	5.69
	0.578	0.604±0.024	104.50	3.97	0.604±0.035	104.50	5.79	0.575±0.020	99.48	3.48
	5.78	5.54±0.21	95.85	3.79	5.53±0.35	95.67	6.33	6.16±0.72	106.57	11.69
GRb_1	2.28	2.20±0.04	96.49	1.82	2.17±0.04	95.18	1.84	2.41±0.38	105.70	15.77
	11.4	10.63±0.15	93.25	1.41	10.97±0.72	96.23	6.56	11.83±0.89	103.77	7.52
	56.9	56.50±0.70	99.30	1.24	51.4±1.7	90.33	3.31	61.27±0.98	107.68	1.60
	228	223.70±8.50	98.11	3.80	215.3±12.1	94.43	5.62	235.1±16.0	103.11	6.81
GRd	0.917	0.863±0.007	94.11	0.81	0.943±0.060	102.84	6.36	0.961±0.157	104.80	16.34
	2.29	2.34±0.01	102.18	0.43	2.39±0.11	104.37	4.60	2.264±0.227	98.86	10.03
	9.17	9.35±1.22	101.96	13.05	8.44±0.17	92.04	2.01	9.037±0.680	98.55	7.52
	91.7	92.99±10.40	101.41	11.18	94.5±9.9	103.05	10.48	97.22±8.70	106.02	8.95
GRg_1	0.0578	0.0575±0.0027	99.48	4.70	0.0546±0.0009	94.46	1.65	0.0596±0.0010	103.11	1.68

	0.144	0.144±0.008	100.00	5.56	0.135±0.012	93.75	8.89	0.141±0.009	97.92	6.38
	0.578	0.577±0.036	99.83	6.24	0.569±0.031	98.44	5.45	0.566±0.048	97.92	8.48
	5.78	5.56±0.33	96.19	5.94	5.91±0.04	102.25	0.68	6.25±0.49	108.13	7.84
GRe	0.0917	0.0902±0.0017	98.36	1.88	0.0870±0.0026	94.87	2.99	0.0944±0.0020	102.94	2.12
	0.229	0.257±0.017	112.23	6.61	0.222±0.014	96.94	6.31	0.221±0.015	96.51	6.79
	0.917	0.926±0.002	100.98	0.22	0.898±0.003	97.93	0.33	0.919±0.057	100.22	6.20
	9.17	8.71±0.39	94.98	4.48	8.66±0.36	94.44	4.16	10.35±0.84	112.87	8.12

For abbreviations of analytes please refer to the “Chemicals and reagents” section.

Table S8: Plasma concentration-time of six target constituents after oral administration of CTE, NGTS, and CNP, respectively.

Time (h)	HSYA (ng·mL^{-1})		GRb$_1$ (ng·mL^{-1})		GRd (ng·mL^{-1})		GRg$_1$ (ng·mL^{-1})		NGR$_1$ (ng·mL^{-1})		GRe (ng·mL^{-1})	
	CTE	CNP	NGTS	CNP	NGTS	CNP	NGTS	CNP	NGTS	CNP	NGTS	CNP
0.083	4.25±1.33	2.58±0.69	17.13±2.84	18.70±4.36	6.19±0.48	6.63±0.64	0.40±0.18	0.38±0.15	0.31±0.12	0.26±0.06	0.25±0.04	0.24±0.02
0.25	7.27±4.48	7.70±2.54	27.95±4.54	22.48±4.27	9.00±2.12	7.13±0.69	0.56±0.15	0.48±0.07	0.46±0.18	0.36±0.05	0.26±0.02	0.25±0.01
0.5	7.34±3.06	9.26±3.38	38.06±3.67	36.06±7.79	11.52±0.97	12.52±4.73	0.37±0.12	0.53±0.37	0.50±0.15	0.53±0.29	0.24±0.02	0.33±0.11
1	8.55±3.78	14.98±4.64	53.50±12.04	45.48±3.29	13.95±4.94	13.52±3.67	0.44±0.19	0.40±0.23	0.45±0.14	0.45±0.19	0.25±0.02	0.24±0.04
1.5	7.97±2.71	7.28±3.10	58.40±12.41	54.60±10.90	14.93±3.51	12.76±1.79	0.33±0.21	0.43±0.32	0.28±0.10	0.33±0.12	0.22±0.02	0.24±0.03
2	5.35±2.07	7.29±3.22	61.92±19.48	60.52±11.99	19.25±6.71	17.58±5.20	0.21±0.05	0.40±0.14	0.25±0.11	0.32±0.05	0.21±0.01	0.23±0.02
3	4.65±2.09	5.13±2.43	78.07±12.75	69.45±9.40	22.33±14.57	18.72±7.48	0.49±0.29	0.53±0.40	0.46±0.20	0.44±0.15	0.27±0.04	0.28±0.10
4	3.07±1.89	3.45±1.91	86.88±41.02	83.87±31.83	23.85±12.58	25.02±15.81	0.34±0.32	0.47±0.13	0.38±0.26	0.38±0.12	0.25±0.05	0.28±0.07
6	2.36±1.60	2.64±0.74	107.10±42.11	112.97±74.57	26.23±10.43	24.85±13.11	0.37±0.13	0.37±0.17	0.25±0.06	0.37±0.17	0.24±0.06	0.23±0.03
8	0.30±0.10	0.35±0.15	75.70±26.34	80.00±20.63	16.68±7.24	15.58±5.29	0.63±0.14	0.65±0.20	0.35±0.06	0.46±0.15	0.13±0.01	0.14±0.03
12	0.25±0.12	0.32±0.17	53.87±19.09	63.88±28.42	8.73±3.74	12.40±7.03	0.59±0.27	0.60±0.36	0.35±0.14	0.38±0.18	–	–
24	–	–	19.18±5.86	22.40±4.86	3.57±1.40	2.85±0.67	0.22±0.13	0.34±0.12	0.14±0.04	0.17±0.06	–	–
48	–	–	18.90±8.97	19.42±3.81	4.15±1.97	3.96±0.34	0.09±0.01	0.13±0.04	–	–	–	–
72	–	–	9.45±3.37	9.62±1.02	2.52±0.63	2.32±0.12	–	–	–	–	–	–
96	–	–	6.79±1.89	6.43±2.45	–	–	–	–	–	–	–	–

For abbreviations of analytes please refer to the "Chemicals and reagents" section.

Table S9: Regression equations, linear ranges, LLOQs and LLODs of the seven metabolites for the cocktail analysis.

Analyte	Regression equation	*r*	Linear range ($pg{\cdot}mL^{-1}$)	LLOQ ($pg{\cdot}mL^{-1}$)	LLOD ($pg{\cdot}mL^{-1}$)
OHOME	$y = 0.00361\,x + 0.00734$	0.9983	1.76 – 881	1.76	0.48
OHBUP	$y = 0.000315\,x + 0.00803$	0.9945	30.4 – 6080	30.40	11.00
OHMID	$y = 0.000561\,x + 0.00535$	0.9967	135 – 27000	9.20	4.80
OHCHL	$y = 0.0206\,x + 0.0773$	0.9944	0.176 – 176	0.55	0.11
OHTOL	$y = 0.00568\,x + 0.0811$	0.9960	13.6 – 6800	4.53	1.08
dEtPHE	$y = 1.74\,x – 0.0119$	0.9944	0.0337 – 2.72	0.034	0.020
dMeDEX	$y = 0.0552\,x + 0.27$	0.9957	3.87 – 1940	1.21	0.50

For abbreviations of analytes please refer to the "Chemicals and reagents" section.

Table S10: Intra- and inter-day precisions and determination accuracies of the seven metabolites for cocktail analysis.

Analyte	Spiked (pg·mL^{-1})	Intra-day (mean ± SD, *n* = 6)			Inter-day (mean ± SD, *n* = 4)		
		Measured (pg·mL^{-1})	Accuracy (%)	Precision (RSD,%)	Measured (pg·mL^{-1})	Accuracy (%)	Precision (RSD,%)
MID	27	26.00±3.94	96.30	15.15	25.67±2.42	95.07	9.43
	135	142.64±11.54	105.66	8.09	132.74±3.40	98.33	2.56
	2710	2813.52±276.53	103.82	9.83	2726.67±215.02	100.62	7.89
	27100	24866.96±2740.35	91.76	11.02	24133.33±1955.33	89.05	8.10
dEtPHE	0.0337	0.0346±0.0018	102.67	5.20	0.0334±0.0006	99.11	1.80
	0.135	0.136±0.005	100.74	3.68	0.137±0.006	101.48	4.38
	0.54	0.55±0.04	101.85	7.27	0.55±0.08	101.85	14.55
	2.70	2.75±0.24	101.85	8.73	2.89±0.22	107.04	7.61
BUP	30.46	28.69±1.45	94.19	5.05	30.38±0.56	99.74	1.84
	60.92	59.70±3.90	98.00	6.53	64.74±3.05	106.27	4.71
	304.6	301.51±17.17	98.99	5.69	305.67±16.50	100.35	5.40
	3046	3021.15±175.12	99.18	5.80	2946.67±66.58	96.74	2.26
dMeDEX	1.21	1.152±0.144	95.21	12.50	1.14±0.03	94.21	2.63
	9.68	9.55±0.64	98.66	6.70	9.71±0.34	100.31	3.50
	387	401.2±4.4	103.67	1.10	395.7±38.3	102.25	9.68
	1940	1674.15±208.95	86.52	12.48	1852.50±265.15	95.74	14.31
OME	4.89	4.71±0.51	96.32	10.72	4.82±0.38	98.57	7.88
	39.1	40.41±3.69	103.35	9.13	41.57±0.95	106.32	2.29
	156	156.81±8.95	100.52	5.71	145.33±6.66	93.16	4.58
	1560	1502.28±64.15	96.30	4.27	1596.67±190.09	102.35	11.91
CHL	0.551	0.53±0.05	96.19	9.43	0.54±0.012	98.00	2.22
	2.2	2.12±0.10	96.36	4.72	2.20±0.06	100.00	2.73
	44.1	46.48±1.42	105.40	3.06	43.60±5.91	98.87	13.56
	176	167.69±12.19	95.28	7.27	166.40±14.64	94.55	8.80
TOL	4.53	4.47±0.22	98.68	4.92	4.32±0.05	95.36	1.16
	17	16.84±0.22	99.06	1.31	17.95±0.35	105.59	1.95
	68	73.17±2.38	107.60	3.25	71.3±0.46	104.85	0.65
	1360	1379.86±123.31	101.46	8.94	1230±45.83	90.44	3.73

For abbreviations of analytes please refer to the "Chemicals and reagents" section.

Table S11: Extraction recoveries and matrix effects of seven target constituents and two IS of cocktail study (mean ± SD, *n* = 3).

Analyte	Spiked (pg·mL^{-1})	Matrix effect (%)	RSD (%)	Recovery (%)	RSD (%)
OHMID	27	91.40꩜7.04	7.70	82.06±2.71	3.31
	135	92.95±12.27	13.2	95.54±2.40	2.51
	2710	102.8±2.44	2.38	94.58±10.30	10.89
	27100	95.39±4.85	5.08	92.87±3.90	4.20
dEtPHE	0.034	85.62±7.32	8.55	106.23±15.73	14.81
	0.27	98.84±14.58	14.75	97.58±11.04	11.32
	1.08	93.45±13.22	14.15	86.16±3.71	4.31
	5.39	104.30±12.65	12.13	99.26±8.94	9.01
OHBUP	60.8	88.34±3.95	4.47	93.14±14.13	15.17
	1520	83.03±7.12	8.55	86.14±2.37	2.76
	3040	102.35±2.79	2.72	89.87±4.66	5.19
	6080	91.48±5.83	6.37	92.11±7.18	7.80
dMeDEX	7.74	96.18±3.89	4.04	102.62±5.93	5.78
	77.4	85.16±5.81	6.82	106.43±1.61	1.51
	387	98.12±4.24	4.32	91.59±9.39	10.25
	3870	91.52±5.87	6.41	79.27±2.67	3.37
OHOME	3.52	102.63±14.2	13.83	91.45±8.88	9.71
	35.2	88.89±2.38	2.68	85.57±5.83	6.81
	88	88.61±13.14	14.83	114.70±3.65	3.18
	1760	100.17±6.14	6.13	92.51±10.44	11.29
OHCHL	0.38	103.40±1.98	1.92	95.14±8.29	8.72
	3.82	112.29±13.97	12.44	89.41±4.89	5.47
	19.1	99.10±3.60	3.63	90.31±3.11	3.44
	191	99.09±5.20	5.24	100.91±1.42	1.4
OHTOL	2.72	98.05±10.25	10.46	96.24±9.43	9.79
	27.2	114.17±12.28	10.76	107.94±7.64	7.08
	1360	96.57±1.38	1.43	98.99±1.86	1.87
	13600	92.24±3.77	4.09	87.73±6.54	7.45
IS2	1590	94.67±2.52	2.66	93.83±2.11	2.25
IS3	27485.85	100.55±1.33	1.32	97.40±3.15	3.23

For abbreviations of analytes please refer to the "Chemicals and reagents" section.

Table S12: *Km* values determined for the enzymatic reaction of the probe substrates and the inhibition IC_{50} values measured for the positive inhibitors to CYP isoforms (mean ± SD, *n* = 3).

CYP	Substrate/metabolite/inhibitor	Tested *Km* (μM)	Reported *Km* (μM)[9]	Tested IC_{50} (μM)	Reported IC_{50} (μM)
3A4	MID/OHMID/KET	10.98 ± 1.51	1 – 14	0.1397 ± 0.0008	0.0037 – 0.18[9]
1A2	PHE/dEtPHE/FUR	65.26 ± 14.35	1.7 – 152	0.5969 ± 0.0514	1.3[9], 1.76 – 13.88[10,11]
2B6	BUP/OHBUP/TRI	70.99 ± 7.45	67 – 168	0.7502 ± 0.0262	1.75 – 26[12,13]
2D6	DEX/dMeDEX/QUI	0.62 ± 0.08	0.44 – 8.5	0.1177 ± 0.0290	0.05[14], 0.0579[11], 0.06[14], 0.12[15]
2C19	OME/OHOME/TIC	7.80 ± 1.65	17 – 26	1.979 ± 0.003	1.2 – 10[9,14]
2E1	CHL/OHCHL/MET	138.6 ± 29.6	39 – 157	0.5116 ± 0.0318	0.83 – 1.6[16]
2C9	TOL/OHTOL/SUL	240.9 ± 48.5	67 – 838	0.1651 ± 0.0159	0.05 – 1[17,18]

For abbreviations of substrates, metabolites, and inhibitors please refer to the "Chemicals and reagents" section.

Table S13: Responses (% control) of HSYA, GRb_1, GRd, GRe, GRg_1, and NGR_1 at their C_{max} levels in the rat plasma.

%Control	CYP2C19	CYP2E1	CYP2C9	CYP2D6	CYP2B6	CYP1A2	CYP3A4
HSYA	77.03	55.38	49.10	47.93	–	–	60.77
GRb_1	98.58	99.99	99.70	98.58	96.46	79.30	99.99
GRd	99.95	98.16	99.97	99.99	–	71.33	85.25
GRe	98.36	97.35	–	–	99.64	99.99	99.99
GRg_1	99.83	99.57	96.27	99.88	98.23	98.46	97.76
NGR_1	92.32	99.99	99.99	92.76	–	–	99.94

For abbreviations of analytes please refer to the "Chemicals and reagents" section of the Supporting information.

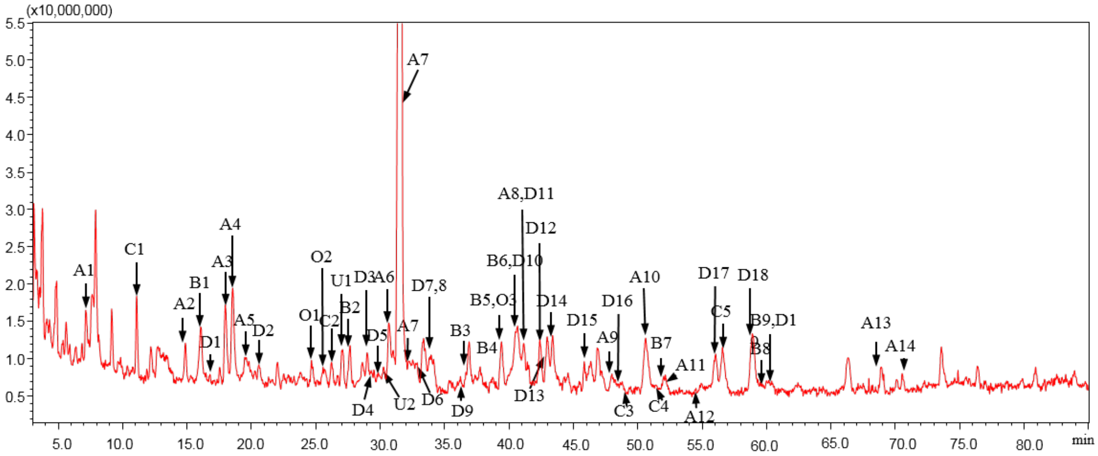

Figure S1. The total ion current chromatogram (TIC) of CTE, the corresponding chemical composition information were reported on the previous researches (Chen, et al., 2014; Analyst 139, 6474–6485).

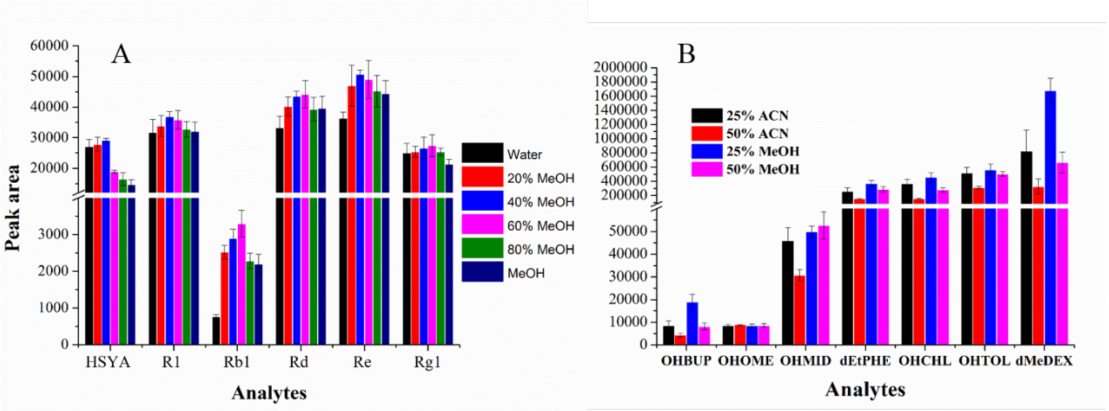

Figure S2. The optimization of sample solvents for the pharmacokinetic analysis (A) and the cocktail assay (B) (n = 3).

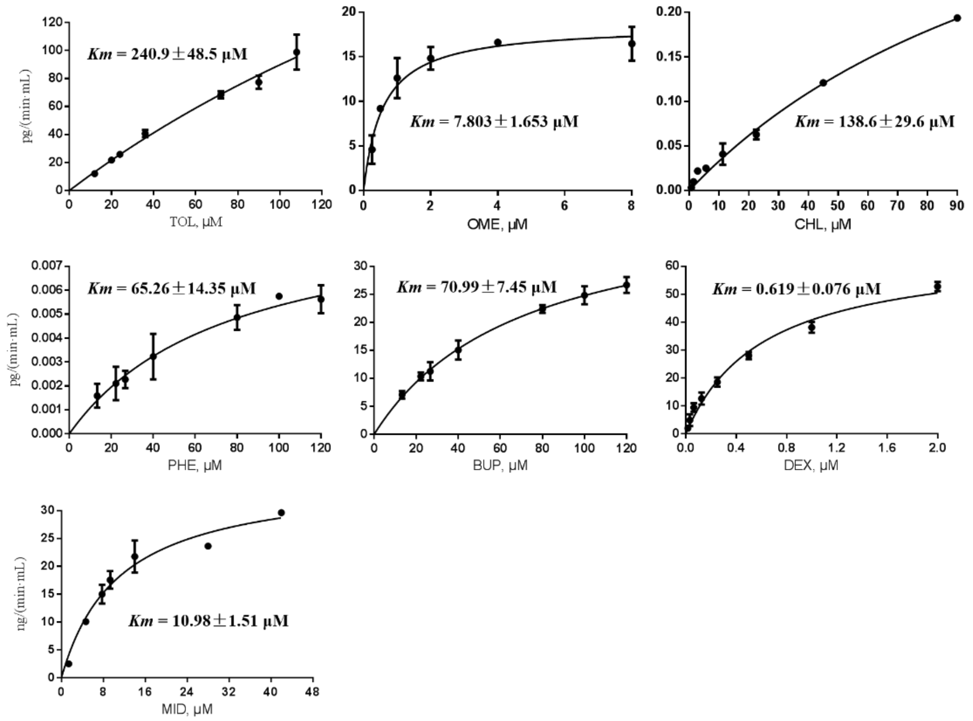

Figure S3. Kinetic profiles for the enzymatic turnover of CYP450-mediated probe reactions.

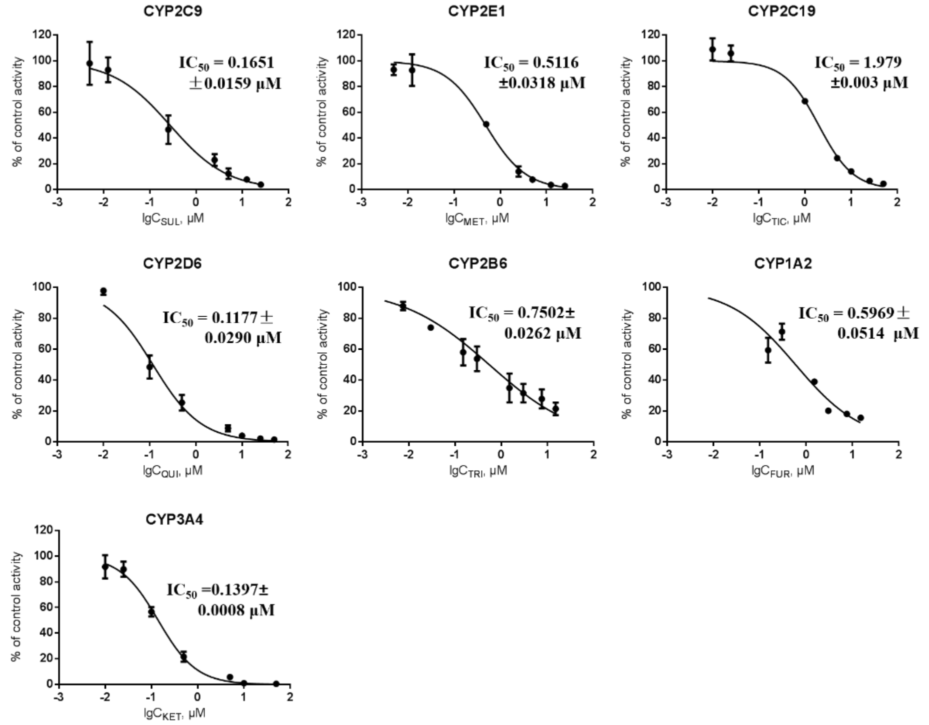

Figure S4. Inhibition curves of the seven positive inhibitors obtained from the substrate cocktail incubation.
